# Processing methods affect phytochemical contents in products prepared from orange‐fleshed sweetpotato leaves and roots

**DOI:** 10.1002/fsn3.2081

**Published:** 2020-12-25

**Authors:** George Ooko Abong', Tawanda Muzhingi, Michael Wandayi Okoth, Fredrick Ng'ang'a, Phillis Emelda Ochieng, Daniel Mahuga Mbogo, Derick Malavi, Machael Akhwale, Sita Ghimire

**Affiliations:** ^1^ Department of Food Science, Nutrition and Technology University of Nairobi Nairobi Kenya; ^2^ Biosciences Eastern and Central Africa International Livestock Research Institute Nairobi Kenya; ^3^ Food and Nutritional Evaluation Laboratory International Potato Centre Nairobi Kenya; ^4^ Kenya Agricultural & Livestock Research Organization (KALRO) Kakamega Centre‐Root and Tuber Crops Program Kakamega Kenya

**Keywords:** dehydration, fermented dehydrated leaves, phytochemicals, processing methods, retention

## Abstract

Phytochemicals enhance human health by acting antagonistically on incidences of cancer and other chronic diseases. They are considered indispensable in a variety of nutraceutical, pharmaceuticals, and medicinal and cosmetic applications. This study evaluated the effects of common processing methods on inherent phytochemical content in the roots and leaves of orange‐fleshed sweetpotato (OFSP) varieties called Kabode and SPK031. Yellosp and Whitesp, which are local sweetpotato varieties, were also included as check for roots and leaves, respectively. The sweetpotato products prepared for phytochemical analysis were boiling roots and leaves, frying chips and crisps, baking bread (for roots only), and fermenting and dehydrating leaves. Phytochemicals that were assessed included vitamin C, total phenolics and flavonoids, tannins, phytates, and soluble oxalates. Results indicated that retention of vitamin C was highest in boiled roots (85%–95%), followed by fries (71%–94%) and crisps (44%–76%), whereas the least retention was in bread (4%–11%) and leaves (0%–27%). Total phenolics, flavonoids, and antioxidant activity in leaves significantly (*p* < .05) varied with the type of processing. Higher retention of these phytochemicals was observed in processed roots but was lowest in bread. Boiling retained more than 100% of all carotenoids, while fermenting and drying the leaves retained 58–62 and 22%–48%, respectively. Frying retained more than 100% of the β‐carotene in the roots, while boiling retained 96%–100%. All processing methods significantly (*p* < .05) reduced antinutrients in leaves and roots. Fermentation of leaves had higher reduction of oxalates, tannins, and phytates, while boiling had the least effect. It is concluded that traditional boiling enhances phytochemical retention in roots but degrades most of them in leaves.

## Graphical abstract

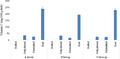

## 1 INTRODUCTION

Root and tuber crops are the second most important source of carbohydrate to humans after cereals and play an important role in ensuring food and nutrition security in the world. Sweetpotato ranks seventh among food crops at global scale and provides food to a large proportion of the world's population (FAOSTAT, 2018). It is a major contributor of energy and phytochemicals in the human diet, the extent of which vary depending on crop variety and processing methods used in diet formulation (Gurmu et al., 2017; Rossi et al., 2016; Shekhar et al., 2015). Orange‐fleshed sweetpotato (OFSP) varieties have been shown to have high nutritional value, particularly in vitamins and minerals compared to many other root and tuber crops. Biofortified OFSP can supply the recommended daily allowance of vitamin A (300–700 μg retinol activity equivalent) for children and lactating women (Rajendran et al., 2017). Both roots and leaves of sweetpotato are commonly utilized as staple food and vegetables by various communities across the world.

Orange‐fleshed sweetpotato roots and leaves are perishable and their postharvest loss is high in developing countries such as Kenya due to limited availability of appropriate storage facilities. As a result, farmers, traders, consumers, and other stakeholders in the sweetpotato value chain experience considerable economic burden. Such losses can be minimized by adopting value addition processes including the production of stable and popular products such as fried sweetpotato crisps, breads, and cakes. However, there is limited information available on the amount and changes in essential phytochemicals (carotenoids, reduced ascorbic acid, phenolics, flavonoids, tannins, phytic acid, and oxalates) in these value‐added products prepared from sweetpotato leaves and roots. The roots of OFSP varieties are generally known for high moisture content, reduced sugars, beta carotene, and other phytochemicals such as flavonoids, vitamin C, phenolics, and anthocyanins. Their quantities in roots can influence the quality and stability of processed products (Anbuselvi & Muthumani, 2014; Chandrasekara & Josheph Kumar, 2016; Swamy & Sinniah, 2015). Similarly, sweetpotato leaves that are used as vegetables by a number of communities contain both phytochemicals and antinutrient factors such as oxalate and tannins. The levels of these constituents in cooked vegetable are affected by the processing method (Abubakar et al., 2010; Min et al., 2012). Previous studies indicated the presence of different phytochemicals in OFSP (Swamy & Sinniah, 2015), but no adequate data were available on quantification of phytochemicals variations in relation to processing popular OFSP varieties in Kenya. Studies have also been conducted on processed crisps and bread from OFSP roots but effect of common deep frying and baking on important OFSP components is not well documented. The stability of crisps and phytochemicals in storage are subjected to oxidative degradation when exposed to light, heat, and air (De Moura et al., 2015b). However, the role of these physical factors during processing on the product stability and phytochemical contents is yet to be established. This study aimed to quantify important phytochemicals in popular food preparation derived from roots and leaves of popular Kenyan OFSP varieties.

## 2 MATERIALS AND METHODS

### 2.1 Source of sweetpotato roots and leaves

Four sweetpotato varieties consisting of two popular OFSP varieties (Kabode and SPK031) and two local varieties (Yellowsp and Whitesp) were used in this study. These varieties were grown in 2018 at the Kenya Agricultural Research Organization (KALRO) in Kakamega following standard cultural practices. All root and leaf samples analyzed in this study were obtained from the crop harvested at maturity. Kabode, SPK031, and Yellowsp were selected based on their popularity in Kenya and their high content of phytochemicals as established in a previous study (Abong' et al., 2020). Whitesp was selected for leaves analysis (instead of SPK031) due to its high yield and wider adoption in Kenya. Further information about these varieties is available at the sweetpotato catalogue (Kapinga et al., 2010). The roots and leaves were harvested, packaged in gunny and net bags, respectively, and transported overnight for analysis to the Biosciences eastern and central Africa‐International Livestock Research Institute (BecA‐ILRI) Hub in Nairobi.

### 2.2 Preparation of raw samples

Approximately 400 g of clean sweetpotato leaves of each variety were weighed and divided into portions of 100 g. The leaves were transferred to Kraft paper bags frozen at −20°C for at least 12 hr and freeze‐dried (Telstar LyoQuest‐55). Similarly, seven roots were randomly selected for each variety, washed with tap water and blotted to dry, and peeled and diced into 0.25 cm cubes. About 400 g of these cubes were divided into portions of 100 g, placed in Kraft paper bags, and frozen overnight at −20°C before freeze drying. Freeze‐dried samples were ground into powder using a Waring Laboratory Blender (Thomas Scientific) and stored at −20°C until analysis.

### 2.3 Preparation of processed samples

Sweetpotato leaves and roots were processed into products that are commonly prepared in Kenya, mimicking local processing parameters in each case. Each food preparation was replicated twice.

#### 2.3.1 Boiling of roots and leaves

Approximately 2 kg of roots from each sweetpotato variety were washed using potable water to remove soil debris, hand‐peeled using kitchen knife, and cut into 5 × 5 cm pieces. The pieces were placed into a cooking pot and potable water added at half the weight before the roots were boiled until a fork could easily penetrate the root. The boiled roots were grated into small pieces, divided into 100 g portions in kraft papers, and deep‐frozen at −20°C for 12 hr before freeze drying.

Sweetpotato leaves (1 kg) were washed to remove surface soil and other foreign matters. The leaves were then cut using a kitchen knife into approximately 5 mm slices. The sliced leaves were placed into a cooking vessel, potable water added at a ratio of 1:1 before being steamed for 20 min until they were confirmed ready for eating. The boiled leaves were further cut into smaller pieces and divided into 100 g portions in kraft papers and deep‐frozen at −20°C for 12 hr before freeze drying.

All freeze‐dried samples were ground using a Warring Laboratory Blender (Thomas Scientific) and stored at −20°C until analysis.

#### 2.3.2 Frying of chips and crisps

About 2 kg roots of each sweetpotato variety were washed using potable water to remove soil debris, hand‐peeled using kitchen knife, and cut into 5 × 5 mm strips for chips and 1.5 mm thickness for crisps. They were deep‐fried in an automatic, temperature‐controlled fryer using Elianto corn oil (Bidco Africa Limited) at 170°C until bubbles ceased. The fries were then removed from oil, drained off excess surface oil, and left to cool. The cooled fries were then grated into small pieces and divided into 100 g portions in kraft papers and deep‐frozen at −20°C for 12 hr before freeze drying. Freeze‐dried samples were ground into powder using a Warring Laboratory Blender (Thomas Scientific) and stored at −20°C until analysis.

#### 2.3.3 Baking wheat–sweetpotato composite bread

Boiled sweetpotato roots for each variety were mashed into puree and incorporated at 40% into wheat flour during the kneading process. The baking procedure was adopted from a previous study (Muzhingi et al., 2018). The bread was then grated into small pieces and divided into 100 g portions in kraft papers and deep‐frozen at −20°C for 12 hr before freeze drying. Freeze‐dried samples were ground into powder using a Warring Laboratory Blender and stored at −20°C until analysis.

#### 2.3.4 Dehydration of leaves

Dehydrated leaves were prepared in two different forms—simple dehydration and lactic acid fermentation followed by dehydration. Sweetpotato leaves (1 kg) were washed to remove surface soil and other foreign matters. The leaves were then blanched in water at 90°C for 3 min before being cut into 5 mm shreds and dried in an air oven at 70°C for 12 hr. Leaves for fermentation were not washed but the process followed sauerkraut fermentation procedure—clean leaves (1 kg) with no visible soil debris were cut into 5 mm shreds and mixed with 2.5% common salt and allowed to ferment anaerobically at prevailing room temperature (24°C) for 7 days for optimal fermentation. The fermented leaves were then dried in an air oven at 70°C for 12 hr. The dried samples were ground into powder using a Warring laboratory electric blender and stored at −20°C until analysis.

### 2.4 Analytical methods

#### 2.4.1 Determination of phytochemicals

Phytochemicals that were determined in raw and cooked sweetpotato roots and leaves were vitamin C, total phenolic compounds, total flavonoids, carotenoids, antioxidants, phytates, tannins, and soluble oxalates. Extraction and subsequent analysis of these phytochemicals was performed following previously established methods (Abong' et al., 2020).

### 2.5 Statistical analysis

Experimental data were analyzed using SAS version 9. Descriptive statistics such as mean and standard deviation of moisture and phytochemical contents were computed. Analysis of variance (ANOVA) was carried out to test for statistical significance among treatments, and the means were separated by Duncan multiple range test at significance level of *p* < .05.

## 3 RESULTS AND DISCUSSION

### 3.1 Vitamin C content in sweetpotato products

**FIGURE 1:**
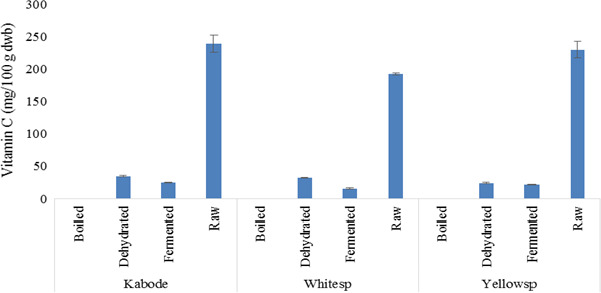
Vitamin C content (dry weight basis) in raw and processed leaves products of three sweetpotato varieties in Kenya. The bars indicate standard error of means

Sweetpotato leaves differed significantly (*p* = .0029) by variety and processing method for vitamin C content, being not detectable in boiled leaves of all varieties and the highest in raw leaves (Figure 1). However, there was no retention of vitamin C in boiled leaves of all varieties, while the highest retention was recorded in dehydrated leaves with retention levels of 24% in Kabode, 27% in Yellowsp, and 10% in Whitesp. Fermented and dehydrated leaves had vitamin C retention at moderate levels (average of 10%).

**FIGURE 2:**
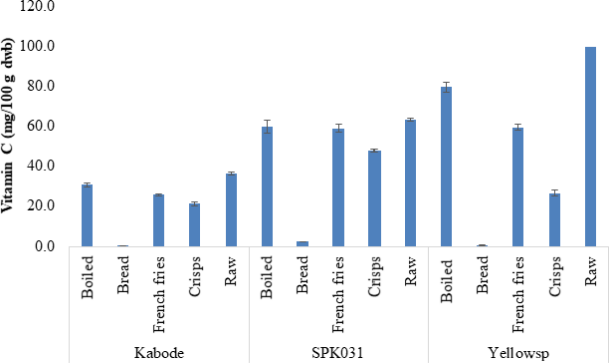
Vitamin C content (dry weight) in roots and processed roots products of three sweetpotato varieties in Kenya. The bars indicate standard error of means

Vitamin C content was highest in boiled roots with an average of 63.46 mg/100 g dry weight basis for the three varieties, followed by chips (54.77 mg/100 g) and crisps (52.94 mg/100 g). The least amount was detected in bread (1.42 mg/100 g) (Figure 2). The levels of vitamin C significantly (*p* < .05) varied with OFSP variety and processing method, with SPK031 variety having the highest retention in boiled roots (95%), chips (94%), and crisps (76%).

Vitamin C content in sweetpotato is controlled by genetic makeup of a variety and cultural practice (Mellidou & Kanellis, 2017). The levels of vitamin C in raw roots in our study were higher than a range of 13.7–23.5 mg/100 g reported in Slovenia (Sinkovič et al., 2017) but comparable to values reported in Tanzania of 273–303 mg/100 g for sweetpotato leaves (Mwanri et al., 2011).Vitamin C is highly susceptible to oxidation more so in aqueous solutions, oxidation being fueled by the presence of oxygen, metal ions such as iron and copper and high temperature (Akah & Onweluzo, 2014; Lee & Kader, 2000). Traditionally, leaves are boiled in water for more than 10 min. Given that the vitamin is soluble in water, its loss is compounded by leaching in boiling water as well as high boiling temperature (Akah & Onweluzo, 2014). The total loss of the vitamin in boiled leaves could have been enhanced by high iron content of the leaves (Lee & Kader, 2000). On the other hand, dehydration led to 80%–90% loss of the vitamin. These results were in agreement with 15% retention reported by other researchers (Mwanri et al., 2011).

On the other hand, bread had a low vitamin C content given that only 40% of OFSP puree was incorporated into wheat to make the bread. In addition, a high temperature under dry heat during baking could have increased the loss of vitamin C. Interestingly, boiled roots retained the highest vitamin C content followed by fries. Even though higher temperature was involved, root matrix could have helped to retain the vitamin, while in fries the vitamin is not leached in oil because it is insoluble in cooking oil. Lower levels of the vitamin in crisps can be attributed to higher surface area to volume ratio that exposed the entire matrix to high frying temperature. Our results to some degree agree with previous studies reporting higher losses of vitamin C in boiled roots (72.37%) and fries (60.9%) (Ikanone & Oyekan, 2014) and 40% loss of vitamin C in boiled roots (Mwanri et al., 2011). However, it was noted that high vitamin C retention in roots (>90%) can be achieved depending on exposure to heat and leaching water (FAO, 2016) as root matrix inhibits excessive loss of the vitamin.

### 3.2 Carotenoid content in processed sweetpotato products

Carotenoids in leaves significantly varied with variety and processing method (Table 1). Lutein was the major carotenoid in leaves, followed by total β‐carotene and zeaxanthin. The levels of these carotenoids in leaves differed significantly (*p* < .05) with processing method. Boiling retained over 100% of all the carotenoids, fermented and dried leaves retained 58%–62%, while dehydrated leaves had the lowest retention (22%–48%). Sweetpotato roots had insignificant amounts of lutein and zeaxanthin carotenoids. The major carotenoid in the roots was the β‐carotene whose derivatives varied significantly with variety and processing methods (Table 2).

**TABLE 1:** Carotenoid contents (dry weight basis) in leaves and processed leaves products of three sweetpotato varieties in Kenya

Variety	Processing method	Lutein	Zeaxanthin	TBC
Kabode	Boiling	60.73 ± 2.80^a^	2.75 ± 0.53^b^	41.27 ± 0.51^a^
	Fermented & dehydrated	20.90 ± 1.97^d^	3.37 ± 0.09^a^	14.97 ± 0.87^e^
	Raw leaves	34.13 ± 2.50^b^	1.08 ± 0.65^e^	22.69 ± 1.84^b^
	Dehydrated	07.73 ± 0.24^h^	0.27 ± 0.01^i^	06.49 ± 0.20^i^
Whitesp	Boiling	35.99 ± 1.02^b^	1.08 ± 0.03^e^	21.36 ± 1.27^b^
	Fermented & dehydrated	09.36 ± 0.31^g^	2.01 ± 0.06^c^	06.06 ± 0.14^j^
	Raw leaves	15.39 ± 1.73^e^	0.29 ± 0.10^h^	11.36 ± 0.65^f^
	Dehydrated	07.34 ± 0.44^h^	0.21 ± 0.02^j^	07.63 ± 0.60^h^
Yellowsp	Boiling	24.28 ± 0.71^c^	1.19 ± 0.04^d^	17.83 ± 0.50^cd^
	Fermented & dehydrated	12.47 ± 0.89^f^	2.13 ± 0.22^c^	09.86 ± 0.48^g^
	Raw leaves	21.53 ± 0.91^d^	0.44 ± 0.03^f^	18.25 ± 0.56^c^
	Dehydrated	06.08 ± 0.55^i^	0.31 ± 0.02^g^	06.04 ± 0.23^j^

Note

Results are means of triplicate samples ± standard deviation; values with similar superscript letters in the same column are not significantly different at *p* ≤ .05.

Abbreviation: TBC, total β‐carotene.

**TABLE 2:** β‐carotene contents (dry weight basis) in roots and processed roots products of three sweetpotato varieties in Kenya

Variety	Processing method	BX	13cisBC	All Trans BC	9 Cis BC	TBC
SPK031	Raw roots	0.55 ± 0.12^f^	0.67 ± 0.15^i^	14.88 ± 1.26^b^	0.53 ± 0.02^ef^	16.63 ± 1.22^cd^
	Boiling	0.27 ± 0.06^i^	1.13 ± 0.04^f^	13.95 ± 0.63^c^	0.54 ± 0.02^ef^	15.89 ± 0.66^de^
	Bread making	0.16 ± 0.08^k^	1.55 ± 0.13^d^	6.52 ± 0.67^f^	0.27 ± 0.01^i^	8.50 ± 0.60^h^
	Chips making	1.19 ± 0.32^b^	6.51 ± 1.07^ab^	26.83 ± 5.26^a^	0.60 ± 0.19^e^	35.13 ± 4.92^a^
	Crisps making	0.80 ± 0.08^c^	7.48 ± 0.88^a^	25.67 ± 3.12^a^	1.07 ± 0.21^d^	35.02 ± 3.11^a^
Kabode	Raw roots	0.60 ± 0.25^e^	0.77 ± 0.30^h^	9.48 ± 4.25^e^	0.24 ± 0.01^j^	11.08 ± 4.00^fg^
	Boiling	0.50 ± 0.01^g^	1.24 ± 0.03^e^	9.38 ± 0.23^e^	0.22 ± 0.02^j^	11.34 ± 0.22^f^
	Bread making	0.20 ± 0.02^j^	0.90 ± 0.16^g^	4.19 ± 0.25^g^	0.15 ± 0.01^l^	5.44 ± 0.24^i^
	Chips making	0.76 ± 0.26^d^	2.94 ± 1.69^c^	14.39 ± 3.77^b^	0.48 ± 0.22^g^	18.57 ± 3.70^c^
	Crisps making	5.12 ± 0.56^a^	0.50 ± 0.04^k^	12.82 ± 1.62^d^	2.43 ± 0.27^a^	20.87 ± 1.60^b^
Yellowsp	Raw roots	ND	0.16 ± 0.01^l^	0.13 ± 0.01^k^	1.21 ± 0.03^c^	1.50 ± 0.03^m^
	Boiling	0.02 ± 0.01^l^	0.12 ± 0.01^m^	0.20 ± 0.03^j^	1.43 ± 0.14^b^	1.78 ± 0.02^l^
	Bread making	0.01 ± 0.00^m^	0.17 ± 0.12^l^	0.49 ± 0.04^i^	0.36 ± 0.06^h^	1.02 ± 0.05^n^
	Chips making	0.25 ± 0.09^i^	0.54 ± 0.01^j^	2.32 ± 0.36^h^	0.17 ± 0.01^k^	3.28 ± 0.30^k^
	Crisps making	0.37 ± 0.06^h^	0.56 ± 0.07^j^	2.12 ± 0.07^h^	0.35 ± 0.02^h^	3.40 ± 0.06^j^

Note

Results are means of triplicate samples ± standard deviation; values with similar superscript letters in the same column are not significantly different at *p* ≤ .05.

Abbreviations: 13CisBC, 13‐cis‐β‐carotene; 9CISBC, 9‐Z‐β‐carotene; All Trans BC, trans‐cis‐β‐carotene; BX, β‐Xanthin; ND, not detected.

Fried sweetpotato root products (chips and crisps) retained over 100% of the β‐carotene, while boiled roots retained 96%–100%. The lowest retention was in bread (48%–68%). The Yellowsp variety that is the most commonly used for roots in Kenya had significantly lower levels of β‐carotene. Carotenoids vary greatly with variety (genetics), plant part, and environment (Chavez et al., 2000; Shekhar et al., 2015). Processing methods influence both the amount and nature of carotenoids. Carotenoids are susceptible to degradation as a result of oxidation or isomerization during the heating process; isomers increase with cooking (Jaeger De Carvalho et al., 2014). In this study, boiling and frying retained over 100% of the carotenoids in leaves and roots. These results contrast with findings of other researchers who reported a decrease in carotenoids with processing (De Moura et al., 2015a; Maziya‐Dixon et al., 2009; Vimala et al., 2011). However, these results agree with Jaeger De Carvalho et al. (2014) who reported an increase of both total carotenoid and carotenoid isomers when pumpkin was boiled in plain and sugary water, as well as in steamed products. Vizzitto and colleagues (Vizzotto et al., 2017) also observed an increase in total carotenoids when twelve genotypes of sweetpotato roots were roasted. Such increase has been attributed to increased availability usually induced by cooking and break down of cell wall structure (cellulose). Cooking disrupts the cell wall, thereby facilitating release of bioactive compounds, making it easy to extract during analysis (Fernández‐García et al., 2012).

### 3.3 Variations in total phenolics, flavonoids, and antioxidant property

Variations in total phenolics, flavonoids and antioxidant properties of sweetpotato leaves and roots are shown in Tables 3 and 4, respectively.

**TABLE 3:** Phenolics, flavonoids, and antioxidants (dry weight basis) in leaves of three sweetpotato varieties in Kenya as influenced by processing methods

Variety	Processing methods	Phenolics (mgGAE/100 g)	Flavonoids (mg CE/100 g)	Antioxidant property (mgTE/100 g)
Kabode	Boiling	1,222.8 ± 58.9^h^	998.2 ± 53.8^i^	551.8 ± 31.2^gh^
	Dehydration	659.3 ± 44.1^i^	411.0 ± 28.8^k^	353.0 ± 35.0^ij^
	Fermentation & dehydration	1,662.4 ± 95.2^g^	913.8 ± 79.8^ij^	574.5 ± 24.8^g^
	Raw	4,451.7 ± 305.5^c^	4,211.2 ± 265.8^a^	1,134.6 ± 49.2^a^
Whitesp	Boiling	3,039.8 ± 219.2^e^	1,554.4 ± 62.2^g^	672.6 ± 7.8^ef^
	Dehydration	3,120.2 ± 318^e^	2,175.9 ± 92.9^d^	698.1 ± 64.9^e^
	Fermentation & dehydration	3,962.3 ± 41.5^d^	3,270.7 ± 48.66^c^	747.3 ± 25.9^d^
	Raw	4,025.4 ± 128.4^d^	1,294.3 ± 112.7^h^	738.7 ± 50.1d^e^
Yellowsp	Boiling	4,699.7 ± 313.3^c^	3,376.5 ± 253.5^c^	372.5 ± 12.5^i^
	Dehydration	5,861.7 ± 496.5^a^	3,816.2 ± 299.9^b^	642.9 ± 51.4^f^
	Fermentation & dehydration	2,280.9 ± 147.9^f^	1,739.2 ± 194.2^f^	878.1 ± 131.4^c^
	Raw	4,999.4 ± 166.5^b^	1,941.2 ± 11.4^e^	1,000.5 ± 18.1^b^

Note

Results are means of triplicate samples ± standard deviation; values with similar superscript letters in the same column are not significantly different at *p* < .05.

**TABLE 4:** Phenolics, flavonoids, and antioxidant (dry weight basis) in roots of three sweetpotato varieties in Kenya as influenced by processing methods

Cultivar	Processing methods	Phenolics (mgGAE/100 g)	Flavonoids (mg CE/100 g)	Antioxidant property (mgTE/100 g)
SPK031	Boiling	184.6 ± 11.8b	78.5 ± 7.9d	36.1 ± 3.2ef
	Bread making	68.5 ± 3.0i	19.5 ± 1.7j	23.4 ± 1.4h
	Chips making	158.5 ± 4.6c	81.6 ± 6.0cd	35.6 ± 5.4ef
	Crisps making	186.9 ± 7.5b	88.6 ± 8.1c	56.3 ± 2.7d
	Raw roots	152.1 ± 4.1cd	53.1 ± 2.6g	78.8 ± 2.0b
Yellowsp	Boiling	200.2 ± 9.9a	58.8 ± 2.1e	62.2 ± 16.3cd
	Bread making	57.0 ± 3.3j	19.8 ± 1.7j	8.7 ± 0.3l
	Chips making	181.6 ± 10.2b	106.4 ± 7.4ab	13.0 ± 0.2k
	Crisps making	195.8 ± 7.9a	111.9 ± 5.5a	35.0 ± 3.2ef
	Raw roots	130.6 ± 5.1ef	19.1 ± 1.0j	93.2 ± 9.8a
Kabode	Boiling	134.7 ± 3.8e	55.8 ± 0.7f	19.0 ± 1.7j
	Bread making	58.2 ± 2.1j	15.9 ± 1.3k	21.1 ± 0.9i
	Chips making	95.6 ± 2.6h	38.4 ± 3.6h	12.9 ± 0.9k
	Crisps making	118.6 ± 5.3g	41.4 ± 3.2h	31.4 ± 1.9g
	Raw roots	97.5 ± 7.4h	28.8 ± 0.9i	37.6 ± 2.1e

Note

Results are means of triplicate samples ± standard deviation; values with similar letters in the same column are not significantly different at *p* < .05.

Phenolics, flavonoids, and antioxidant property in sweetpotato leaves varied significantly (*p* < .05) with processing method. Even though the lowest retention was noted in dehydrated leaves, there was no defined trend of retention in these chemical properties. Whereas total phenolics was reduced with processing, phenolics seemed to be retained beyond 100% by most processing methods that can be attributed to ease of extraction in processed leaves whose tissues had been raptured in the process (Curayag et al., 2019). Antioxidant activity that indicate the ability of phytochemicals to protect the body against autoxidation was reduced in all processing methods mainly because it is contribution of other chemical constituents such as vitamin C that decreased with processing. It is important to note that fermented and dehydrated leaves had enhanced antioxidant property as also observed by other researchers (Adetuyi & Ibrahim, 2014).

On the other hand, boiling and frying of the roots retained phenolics and flavonoids over 100%. This can be explained by the fact that the heating process raptures the plant cell wall, making available inherent contents at high levels (Jaeger De Carvalho et al., 2014). In this study, Kabode (most common OFSP in Kenya) had the lowest antioxidant activity compared to Yellowsp, a yellow‐fleshed variety that had quite a low level of β‐carotene. This shows that apart from the carotenoids, other factors contribute to antioxidative property of sweetpotato roots that should all be enhanced for optimum benefit of the consumer. These results are contrary to findings by other authors who reported an increase in antioxidant activity of dehydrated purple sweetpotato (Curayag et al., 2019).

### 3.4 Antinutritional factors in processed sweetpotato leaf and root products

Variations in soluble oxalates, tannins, and phytic acid were significantly (*p* < .05) affected by processing method, variety, and plant part as shown in Tables 5 and 6, respectively. All processing methods reduced the antinutrients to different extents. In leaves, lactic acid fermentation, followed by dehydration, reduced the antinutrients the most. For instance, oxalates were reduced by 27%–36%, tannins by 34%–65%, and phytic acid by 50%–77%. Simply dehydrating the leaves led to a reduction of oxalates (3%–27%), tannins (35%–62%), and phytic acid (36%–56%), while the lowest reduction was in boiled leaves at 0.5%–23% for oxalates, 31%–59% for tannins, and 16%–30% for phytic acid. These antinutritional factors limit bioavailability of minerals and assimilation of proteins. For instance in phytates, the complex phosphorus makes phytic phosphorus unavailable (Ahmad et al., 2013). Boiling and dehydration rapture cell walls leading to leaching and degradation of antinutrients. However, fermentation has been shown to break down antinutrients through microbial action, thereby reducing their negative effect (Adane et al., 2013).

**TABLE 5:** Antinutritional factors (dry weight basis) in leaves of three sweetpotato varieties in Kenya as influenced by processing methods

Variety	Processing method	Oxalates (mg/100g)	Tannins (g/100 g)	Phytic acid (g/100 g)
Kabode	Boiling	1,399.67 ± 16.72^e^	2.85 ± 0.30^h^	0.41 ± 0.01^d^
	Dehydration	1,321.90 ± 258.51^f^	2.69 ± 0.01^hi^	0.30 ± 0.28^de^
	Fermentation & dehydration	1,325.13 ± 181.28^f^	3.84 ± 0.14^g^	0.13 ± 0.09^h^
	Raw	1,820.56 ± 53.45^a^	6.95 ± 0.42^bc^	0.58 ± 0.17^bc^
Whitesp	Boiling	1,468.80 ± 344.77^d^	5.35 ± 0.21^d^	0.74 ± 0.54^b^
	Dehydration	1,690.56 ± 27.63^c^	5.08 ± 0.48^de^	0.67 ± 0.16^b^
	Fermentation & dehydration	1,107.36 ± 174.75^gh^	4.94 ± 0.14^e^	0.49 ± 0.15^d^
	Raw	1,737.20 ± 70.69^b^	7.81 ± 0.99^b^	1.07 ± 0.18^a^
Yellowsp	Boiling	1,341.63 ± 25.22^ef^	6.67 ± 0.25^c^	0.46 ± 0.24^d^
	Dehydration	1,312.50 ± 283.59^fg^	7.32 ± 0.43^bc^	0.24 ± 0.20^fg^
	Fermentation & dehydration	913.96 ± 109.97^h^	4.19 ± 0.14^f^	0.28 ± 0.07^f^
	Raw	1,347.66 ± 69.84^ef^	11.79 ± 0.57^a^	0.55 ± 0.26^bc^

Note

Results are means of triplicate samples ± standard deviation; values with similar superscript letters in the same column are not significantly different at *p* ≤ .05.

**TABLE 6:** Antinutritional factors (dry weight basis) in roots of three sweetpotato varieties in Kenya as influenced by processing methods

Variety	Processing method	Oxalates (mg/100g)	Tannins (g/100 g)	Phytic acid (g/100 g)
Kabode	Boiling	164.48 ± 6.49^cd^	0.15 ± 0.00^f^	0.61 ± 0.22^cb^
	Raw roots	217.35 ± 7.60^b^	0.24 ± 0.02^b^	0.85 ± 0.39^ab^
	Bread making	66.61 ± 1.13^j^	0.12 ± 0.01^g^	0.24 ± 0.02^ef^
	Chips making	124.88 ± 6.60^f^	0.18 ± 0.01d^e^	0.80 ± 0.31^b^
	Crisps making	186.71 ± 23.01^c^	0.23 ± 0.04^c^	0.23 ± 0.09^ef^
SPK	Boiling	129.83 ± 6.56^f^	0.20 ± 0.02^d^	0.68 ± 0.34^cb^
	Raw roots	181.36 ± 4.94^c^	0.28 ± 0.06^a^	0.95 ± 0.37^a^
	Bread making	83.18 ± 3.99^h^	0.19 ± 0.02^d^	0.30 ± 0.11^e^
	Chips making	96.72 ± 3.17^g^	0.24 ± 0.01^b^	0.85 ± 0.29^ab^
	Crisps making	127.33 ± 10.76^f^	0.25 ± 0.02^ab^	0.38 ± 0.32^ed^
Yellow	Boiling	184.27 ± 17.39^c^	0.12 ± 0.04^g^	0.25 ± 0.27^ef^
	Raw roots	223.97 ± 18.39^a^	0.32 ± 0.15^a^	0.56 ± 0.30^cd^
	Bread making	77.13 ± 2.61^i^	0.12 ± 0.01^g^	0.23 ± 0.02^ef^
	Chips making	139.80 ± 3.81^e^	0.19 ± 0.01^d^	0.40 ± 0.25^cd^
	Crisps making	134.32 ± 6.28^ef^	0.23 ± 0.03^c^	0.11 ± 0.04^g^

Note

Results are means of triplicate samples ± standard deviation; values with similar superscript letters in the same column are not significantly different at *p* ≤ .05.

Sweetpotato roots had lower concentrations of the antinutrients compared to leaves. All processing methods significantly reduced the antinutrients and they were consistently lower in bread given that sweetpotato was used to substitute wheat at 40% in which case the reduction in all these factors was more than 50%. Frying decreased the antinutrient oxalates and phytic acid more than simply boiling the roots. On the other hand, boiling roots reduced tannins more than frying. The current values of reduction in antinutrients in sweetpotato are lower compared to what other researchers have reported for sweetpotato and other roots (Oluwatoyin & Oreoluwa, 2019).

## 4 CONCLUSION

This study documents effect of processing methods and the interaction between processing methods and sweetpotato varieties on inherent phytochemical content of processed foods prepared from leaves and roots of selected sweetpotato varieties in Kenya. We conclude that traditional boiling enhances the retention of phytochemicals in roots, whereas boiling degrades most of these phytochemicals in leaves. Fermentation and dehydration of leaves and frying sweetpotato roots leads to reduction of antinutrients in sweetpotato leaves and roots, respectively. This study shows the possibility of selecting processing method to improve the target phytochemical content in the processed sweetpotato food products.

## CONFLICT OF INTEREST

The authors declare they do not have any conflict of interest.

## ETHICAL APPROVAL

This study does not involve any human or animal testing.

## References

[fsn32081-bib-0001] Abong', G. , Muzhingi, T. , Wandayi Okoth, M. , Ng'ang'a, F. , Ochieng', P. E. , Mahuga Mbogo, D. , Malavi, D. , Akhwale, M. , & Ghimire, S. (2020). Phytochemicals in Leaves and Roots of Selected Kenyan Orange Fleshed Sweet Potato (OFSP) Varieties. International Journal of Food Science, 2020, 1–11. 10.1155/2020/3567972 PMC7007951 32083118

[fsn32081-bib-0002] Abubakar, H. , Olayiwola, I. , Sanni, S. , & Idowu, M. (2010). Chemical composition of sweet potato (Ipomea batatas Lam) dishes as consumed in Kwara state, Nigeria. International Food Research Journal, 17, 411–416.

[fsn32081-bib-0003] Adane, T. , Shimelis, A. , Negussie, R. , Tilahun, B. , & Haki, G. (2013). Effect of processing method on the proximate composition, mineral content and antinutritional factors of taro (Colocasia esculenta, L.) grown in Ethiopia. African Journal of Food, Agriculture, Nutrition and Development, 13(2), 7383–7398. http://www.bioline.org.br/pdf?nd13023

[fsn32081-bib-0004] Adetuyi, F. O. , & Ibrahim, T. A. (2014). Effect of fermentation time on the phenolic, flavonoid and vitamin C contents and antioxidant activities of Okra (Abelmoschus esculentus) seeds. Nigerian Food Journal, 32(2), 128–137. 10.1016/S0189-7241(15)30128-4

[fsn32081-bib-0005] Ahmad, I. , Mohammad, F. , Zeb, A. , Noorka, I. R. , Farhatullah, S. , & Jadoon, S. A. (2013). Determination and inheritance of phytic acid as marker in diverse genetic group of bread wheat. American Journal of Molecular Biology, 3(July), 158–164. 10.4236/ajmb.2013.33021

[fsn32081-bib-0006] Akah, N. P. , & Onweluzo, J. C. (2014). Evaluation of water‐soluble vitamins and optimum cooking time of fresh edible portions of Elephant Grass (Pennisetum purpureum L. Schumach) Shoot. Nigerian Food Journal, 32(2), 120–127. 10.1016/S0189-7241(15)30127-2

[fsn32081-bib-0007] Anbuselvi, S. , & Muthumani, S. (2014). Phytochemical and antinutritional constituents of sweet potato. Journal of Chemical and Pharmaceutical Research, 6(2), 380–383.

[fsn32081-bib-0008] Chandrasekara, A. , & Josheph Kumar, T. (2016). Roots and tuber crops as functional foods: a review on phytochemical constituents and their potential health benefits. International Journal of Food Science, 2016, 1–15. 10.1155/2016/3631647 PMC4834168 27127779

[fsn32081-bib-0009] Chavez, A. L. , Bedoya, J. M. , Sánchez, T. , Iglesias, C. , Ceballos, H. , & Roca, W. (2000). Iron, carotene, and ascorbic acid in cassava roots and leaves. Food and Nutrition Bulletin, 21(4), 410–413. 10.1177/156482650002100413

[fsn32081-bib-0010] Curayag, Q. A. L. , Dizon, E. I. , Hurtada, W. A. , Ann, Q. , Dizon, E. I. , & Hurtada, W. A. (2019). Antioxidant activity, chemical and nutritional properties of raw and processed purple‐fleshed sweet potato (Ipomoea batatas Lam.). Cogent Food and Agriculture, 1932, 1–14. 10.1080/23311932.2019.1662930

[fsn32081-bib-0011] De Moura, F. F. , Miloff, A. , & Boy, E. (2015a). Retention of provitamin a carotenoids in staple crops targeted for biofortification in Africa: Cassava, maize and sweet potato. Critical Reviews in Food Science and Nutrition, 55(9), 1246–1269. 10.1080/10408398.2012.724477 24915386 PMC4353306

[fsn32081-bib-0012] De Moura, F. F. , Miloff, A. , & Boy, E. (2015b). Retention of provitamin A carotenoids in staple crops targeted for biofortification in Africa: Cassava, maize and sweet potato. Critical Reviews in Food Science and Nutrition, 55(9), 1246–1269. 10.1080/10408398.2012.724477 24915386 PMC4353306

[fsn32081-bib-0013] FAO (2016). Roots, tubers, plantains and bananas in human nutrition ‐ Effect of processing on nutritional value. Technical Report. Retrieved from http://www.fao.org/docrep/t0207e/T0207E07.htm Rome, Italy: United Nation http://www.fao.org/3/t0207e/T0207E00.htm.

[fsn32081-bib-0014] FAOSTAT (2018). Most produced commodities worldwide. In Food and Agriculture Organization (FAO). Retrieved from http://www.fao.org/faostat/es/#compare Rome, Italy: United Nation.

[fsn32081-bib-0015] Fernández‐García, E. , Carvajal‐Lérida, I. , Jarén‐Galán, M. , Garrido‐Fernández, J. , Pérez‐Gálvez, A. , & Hornero‐Méndez, D. (2012). Carotenoids bioavailability from foods: From plant pigments to efficient biological activities. Food Research International, 46(2), 438–450. 10.1016/j.foodres.2011.06.007

[fsn32081-bib-0016] Gurmu, F. , Hussein, S. , & Laing, M. (2017). Genotype‐by‐environment interaction and stability of sweetpotato genotypes for root dry matter, β‐carotene and fresh root yield. Open Agriculture, 2(1), 473–485. 10.1515/opag-2017-0052

[fsn32081-bib-0017] Ikanone, C. E. O. , & Oyekan, P. O. (2014). Effect of boiling and frying on the total carbohydrate, vitamin C and mineral contents of Irish (Solanun tuberosum) and Sweet (Ipomea batatas) potato tubers. Nigerian Food Journal, 32(2), 33–39. 10.1016/S0189-7241(15)30115-6

[fsn32081-bib-0018] Jaeger De Carvalho, L. M. , De, L. , Sarmet, A. , Smiderle, M. , Luiz, J. , Carvalho, V. D. , De Souza, F. , Cardoso, N. , Gabriela, M. , & Koblitz, B. (2014). Assessment of carotenoids in pumpkins after different home cooking conditions. Food Science and Technology (Campinas), 34(2), 365–370. 10.1590/fst.2014.0058

[fsn32081-bib-0019] Kapinga, R. , Tumwegamire, S. , Ndunguru, J. , Andrade, M. I. , Agili, S. , Mwanga, R. O. , Laurie, S. , & Dapaah, H. (2010). Catalogue of orange‐fleshed sweetpotato varieties for Sub‐Saharan Africa. International Potato Center (CIP) https://cipotato.org/wp‐content/uploads/2014/08/005374.pdf

[fsn32081-bib-0020] Lee, S. K. , & Kader, A. A. (2000). Preharvest and postharvest factors influencing vitamin C content of horticultural crops. Postharvest Biology and Technology, 20, 207–220.

[fsn32081-bib-0021] Maziya‐Dixon, B. , Dixon, A. G. O. , & Ssemakula, G. (2009). Changes in total carotenoid content at different stages of traditional processing of yellow‐fleshed cassava genotypes. International Journal of Food Science & Technology, 44(12), 2350–2357. 10.1111/j.1365-2621.2007.01638.x

[fsn32081-bib-0022] Mellidou, I. , & Kanellis, A. K. (2017). Genetic control of ascorbic acid biosynthesis and recycling in horticultural crops. Frontiers in Chemistry, 5, 1–8. 10.3389/fchem.2017.00050 28744455 PMC5504230

[fsn32081-bib-0023] Min, Y. , Hyun, J. , Bong, J. , Young, S. , Nam, M. , Young, M. , Sook, J. , Kim, J. , & Hyun, J. (2012). Changes in the physiological activities of four sweet potato varieties by cooking condition. The Korean Nutrition Society, 45(1), 12–19. 10.4163/kjn.2012.45.1.12

[fsn32081-bib-0024] Muzhingi, T. , Owade, J. O. , Abong, G. O. , Okoth, M. W. , Heck, S. , Low, J. , Mbogo, D. , & Malavi, D. (2018). Sensory attributes of composite breads from shelf storable orange‐fleshed sweetpotato puree. Open Agriculture, 3, 459–465. 10.1515/opag-2018-0051

[fsn32081-bib-0025] Mwanri, A. , Kogi‐Makau, W. , & Laswai, S. (2011). Nutrient and Antinutrients composition of raw, cooked and sun‐dried sweetpotato leaves. African Journal of Food, Agriculture, Nutrition and Development, 1(5), 5142–5156.

[fsn32081-bib-0026] Oluwatoyin, A. , & Oreoluwa, F. (2019). Processing of some tubers and the effect on the carbohydrate digestion, functional groups and the morphology of the extracted starches. Journal of Integrative Food Sciences and Nutrition, 2(1), 1–9.

[fsn32081-bib-0027] Rajendran, S. , Kimenye, L. N. , & McEwan, M. (2017). Strategies for the development of the sweetpotato early generation seed sector in eastern and southern Africa. Open Agriculture, 2(1), 236–243. 10.1515/opag-2017-0025

[fsn32081-bib-0028] Rossi, S. , Tubiello, F. N. , Prosperi, P. , Salvatore, M. , Jacobs, H. , Biancalani, R. , House, J. I. , & Boschetti, L. (2016). Estimates of greenhouse gas emissions from biomass and peat fires. Climatic Change, 135(3–4), 699–711. 10.1007/s10584-015-1584-y

[fsn32081-bib-0029] Shekhar, S. , Mishra, D. , Buragohain, A. K. , Chakraborty, S. , & Chakraborty, N. (2015). Comparative analysis of phytochemicals and nutrient availability in two contrasting cultivars of sweet potato (Ipomoea batatas L.). Food Chemistry, 173, 957–965. 10.1016/j.foodchem.2014.09.172 25466112

[fsn32081-bib-0030] Sinkovič, L. , Pipan, B. , Meglič, V. , Kunstelj, N. , Nečemer, M. , Zlatić, E. , & Žnidarčič, D. (2017). Genetic differentiation of Slovenian sweet potato varieties (Ipomoea batatas) and effect of different growing media on their agronomic and nutritional traits. Italian Journal of Agronomy, 11, 1–25. 10.4081/ija.2017.949

[fsn32081-bib-0031] Swamy, M. K. , & Sinniah, U. R. (2015). A comprehensive review on the phytochemical constituents and pharmacological activities of Pogostemon cablin Benth.: An aromatic medicinal plant of industrial importance. Molecules, 20(5), 8521–8547. 10.3390/molecules20058521 25985355 PMC6272783

[fsn32081-bib-0032] Vimala, B. , Nambisan, B. , & Hariprakash, B. (2011). Retention of carotenoids in orange‐fleshed sweet potato during processing. Journal of Food Science and Technology, 48(4), 520–524. 10.1007/s13197-011-0323-2 23572783 PMC3551189

[fsn32081-bib-0033] Vizzotto, M. , Pereira, E. D. S. , Vinholes, J. R. , Munhoz, P. C. , Ferri, N. M. L. , Castro, L. A. S. D. , & Krolow, A. C. R. (2017). Physicochemical and antioxidant capacity analysis of colored sweet potato genotypes: In natura and thermally processed. Ciência Rural, 47(4), 1–8. 10.1590/0103-8478cr20151385

